# 3D super-resolution deep-tissue imaging in living mice

**DOI:** 10.1364/OPTICA.416841

**Published:** 2021-03-25

**Authors:** Mary Grace M. Velasco, Mengyang Zhang, Jacopo Antonello, Peng Yuan, Edward S. Allgeyer, Dennis May, Ons M’Saad, Phylicia Kidd, Andrew E. S. Barentine, Valentina Greco, Jaime Grutzendler, Martin J. Booth, Joerg Bewersdorf

**Affiliations:** 1 Department of Biomedical Engineering, Yale School of Engineering & Applied Science, New Haven, Connecticut 06520, USA; 2 Department of Cell Biology, Yale School of Medicine, New Haven, Connecticut 06520, USA; 3 Interdepartmental Neuroscience Program, Yale School of Medicine, New Haven, Connecticut 06520, USA; 4 Department of Neuroscience, Yale School of Medicine, New Haven, Connecticut 06520, USA; 5 Department of Engineering Science, University of Oxford, Oxford OX1 3PJ, UK; 6 Department of Neurology, Yale School of Medicine, New Haven, Connecticut 06520, USA; 7 Current Address: Department of Biology, Stanford University, Stanford, California 94304, USA; 8 Current Address: The Gurdon Institute, University of Cambridge, Cambridge CB21QN, UK; 9 Department of Genetics, Yale School of Medicine, New Haven, Connecticut 06520, USA; 10 Department of Dermatology, Yale Stem Cell Center, Yale Cancer Center, Yale School of Medicine, New Haven, Connecticut 06520, USA

## Abstract

Stimulated emission depletion (STED) microscopy enables the three-dimensional (3D) visualization of dynamic nanoscale structures in living cells, offering unique insights into their organization. However, 3D-STED imaging deep inside biological tissue is obstructed by optical aberrations and light scattering. We present a STED system that overcomes these challenges. Through the combination of two-photon excitation, adaptive optics, red-emitting organic dyes, and a long-working-distance water-immersion objective lens, our system achieves aberration-corrected 3D super-resolution imaging, which we demonstrate 164 µm deep in fixed mouse brain tissue and 76 µm deep in the brain of a living mouse.

## 1. INTRODUCTION

Much like the invention of the light microscope itself, the advent of super-resolution fluorescence microscopy has triggered paradigm shifts in the way we study biology [1]. In an era where imaging resolution is no longer dictated by Abbe’s law of diffraction [2], the noninvasive visualization of nanoscale dynamic structures in living cells is now attainable [3,4]. Of the super-resolution imaging modalities, stimulated emission depletion (STED) microscopy [5] is most readily extended to imaging thick tissue samples. The confocal pinhole integrated into most STED microscopes provides an optical sectioning effect that has enabled super-resolution imaging in fruit flies [6], whole worms [7], and even living mice [8,9].

The typically ring-shaped depletion focus of a STED microscope is most often generated by applying a 0 to 2π azimuthal phase ramp (or “vortex”) to the depletion beam [10] using either a static phase plate [11] or, more recently, a spatial light modulator (SLM) [12]. Unfortunately, the depletion effects of this focus are limited to the lateral (x−y) direction, such that the effective point spread function (PSF) remains diffraction limited in the axial (z) direction.

To improve the axial resolution of STED microscopy, two methods have proven useful. First, the STED principle can be combined with a 4Pi geometry featuring two opposing objective lenses [13]. 4Pi-STED systems have been able to achieve three-dimensional (3D) isotropic ∼30nm super-resolution [14], earning them the name “isoSTED” [15]. However, isoSTED microscopes, in addition to being optically complex, are also typically limited to thin samples. Moreover, the need for opposing objectives precludes their application in larger specimens such as living mice.

An alternative approach to 3D-STED microscopy is the use of a single-objective geometry with a radially symmetric “top-hat” depletion phase mask, so named for the central π-step [16]. The top-hat phase mask generates a depletion focus with two high-intensity lobes above and below the focal plane for fluorescence depletion along the optical axis. While the axial resolution achieved in this way does not match that of an isoSTED microscope, the single-objective geometry permits the imaging of thick samples, including living mice. Furthermore, the top-hat phase mask can be combined with a traditional vortex phase mask to overcome the weak lateral depletion achieved with the top-hat focus alone [17].

3D-STED microscopes that exploit the top-hat phase mask still find limited application in deep-tissue imaging experiments. This is primarily due to the susceptibility of the top-hat depletion focus to optical aberrations [12,18–20], which arise from refractive index mismatches between the objective lens immersion medium and the specimen. If left uncorrected, aberrations can raise the intensity minimum in the center of the depletion focus, causing fluorescence emission to be depleted entirely, rather than just being confined to the center of the depletion ring. Most STED microscopes rely on oil (e.g., [3]) or glycerol (e.g., [9,21]) immersion objective lenses due to their high NAs, which promise better resolution. However, the 3D-STED imaging of aqueous living specimens using these lenses can prove challenging due to the refractive index of the immersion medium being too high. Recently, Heine *et al.* replaced the oil or glycerol immersion objective lens of a typical STED microscope with a water-immersion objective to minimize the spherical aberration induced when imaging aqueous living specimens using 3D-STED [22]. Despite the lower NA of water-immersion objectives, the authors resolved 153 nm structures axially. Nevertheless, the imaging depths achieved were still limited by scattering, the objective working distance, and specimen-induced aberrations (in addition to spherical aberration) that remained uncorrected.

To address scattering, super-resolution microscopy can be combined with two-photon excitation (2PE), which utilizes near-infrared (NIR) excitation wavelengths and is the preferred modality for imaging deep in scattering tissue [23]. 2PE has previously been combined with structured illumination microscopy (SIM) for this reason [24], though its combination with STED microscopy [25–28] has the added advantage of sub-100 nm resolution capabilities without any image processing requirement. Moreover, recent developments in STED microscopy have exploited robust red- and far-red-emitting organic dyes, which, in addition to being excitable via 2PE [29], also require NIR depletion wavelengths. The shift of both the excitation and depletion light to the NIR regime allows for deeper penetration in scattering tissue, making this configuration ideal for deep-tissue imaging.

The presence of optical aberrations is still a major concern for deep-tissue 3D-STED microscopy, even when it is coupled with 2PE. Biological tissue can be very optically heterogeneous, and the refractive index is typically highly varying in space. While clearing the sample can address this issue and make the sample more homogenous [30], clearing methods are not compatible with living specimens. A gentler approach that is nondestructive to the sample is the use of objective lens correction collars, which can compensate for spherical aberrations [31,32]. However, correction collars are difficult to adjust on the fly, and they only correct for spherical aberrations while leaving the other aberration modes uncorrected. An alternative method for aberration correction that is equally gentle on specimens is the use of adaptive optics (AO) [33]. In AO, a corrective element such as an SLM or a deformable mirror (DM) is imaged onto the back focal plane of the objective lens and is programmed to impart a phase variation that is equal but opposite to that induced by the sample. The cumulative phase at the sample plane is thus zero, and aberration-free excitation and depletion foci can be recovered. This active approach to aberration correction is ideal for imaging biological specimens where complex, highly varying refractive index maps are the norm [34,35].

Previous implementations of AO in 3D-STED microscopy have determined the corrective phase modulation using a metric-based approach [12,36–38]. The applied correction is systematically adjusted to optimize the quality of the final image. For a given aberration mode, a sequence of images is acquired, with each image corresponding to a different mode coefficient. Each image is then quantified using an image quality metric, and the optimal amount of correction is estimated as that which maximizes this metric. This approach, when combined with 3D-STED microscopy, has enabled aberration-corrected imaging of the complete 15 µm thick mitotic spindle in fixed cells [37] and of fluorescent beads through 25 µm of fixed zebrafish retina tissue [12].

Alternatively, wavefront sensing (WFS) can be used to directly measure the sample-induced aberrations. Conveniently, in 2PE microscopes, the guide star required for this approach is provided by the two-photon-excited fluorescent volume [39,40], which is inherently confined in three dimensions. Fluorescence from this “nonlinear guide star” is descanned and directed to a Shack–Hartman sensor (SHS). The SHS then samples the wavefront using a microlens array and generates a spot diagram on a camera positioned at the focal plane of the microlenses. If the wavefront is aberrated, the spots will be displaced from the center of their designated subregions. Using these displacements, the SHS can reconstruct the wavefront and determine its constituent Zernike modes and thus the corrective phase that needs to be added to the system. This approach to aberration correction has been used to recover the optimal resolution of 2PE laser scanning [39,40] and SIM [41,42] microscopes deep in tissue, including in a living mouse [42,43].

Here we present a 2PE-STED microscope capable of 3D subdiffraction-limit resolution deep in aberrating tissue. Its capabilities are made possible by the combined effect of 2PE, red-emitting organic dyes, WFS-based aberration correction, and a long-working-distance water-immersion objective lens. We demonstrate the system’s capabilities by visualizing the 3D chromatin structure of keratinocytes in fixed mouse skin tissue. We then demonstrate 3D-STED imaging in living mice, using a labelling strategy that enables neuronal labeling of the intact mouse brain with ATTO590, a photostable, live-cell and STED-compatible organic dye [44].

## 2. RESULTS

### A. Optical Setup

**Fig. 1.**
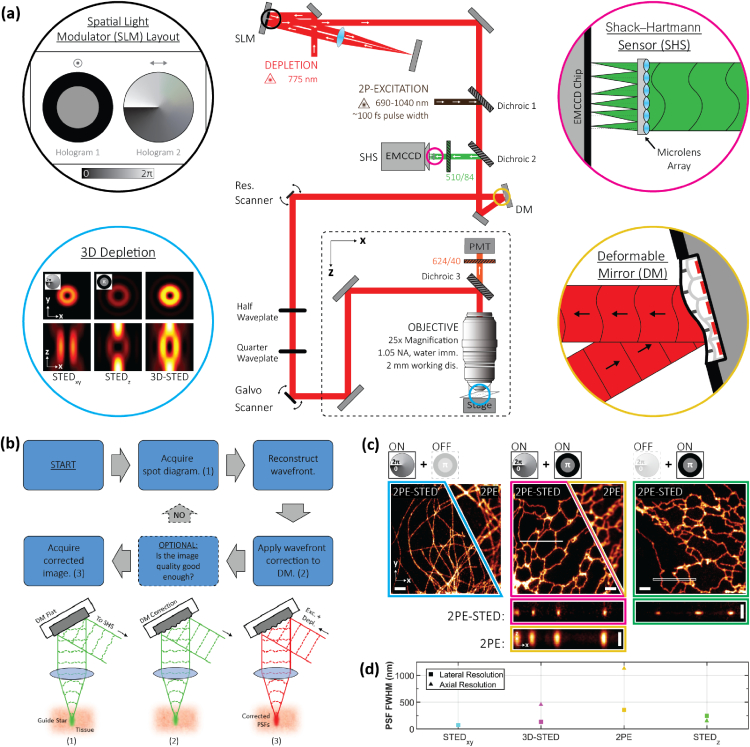
Principles of aberration-corrected 3D-2PE-STED microscopy. (a) Simplified schematic of the optical setup. Detailed schematic is available in Fig. S1 in Supplement 1. (b) Aberration-correction routine. The routine consists of three steps: (1) guide star generation and wavefront reconstruction, (2) aberration correction, and (3) super-resolution image acquisition. See main text for details. (c) 2PE and 2PE-STED images of ATTO594-labelled microtubules (left) and ER tubules (middle and right) in fixed COS7 cells. Depletion via either or both the vortex and top-hat phase masks was used, as indicated by the schematic above each image. A 3D Gaussian blur of $\sigma_{\text{xy}}=39\;nm$ and $\sigma_{z}=50\;nm$ was applied to all images; x−y images depict either a single frame (cyan) or a maximum intensity projection (MIP) of an image stack (magenta and green); x−z images depict MIPs of the regions demarcated by the white box in the corresponding x−y image, resliced in the x−z direction. Scale bars are 2 µm in the x−y images and 1 µm in the x−z images. (d) Plots summarizing the lateral and axial resolution of the system. See main text and Supplement 1 for details. Data points are colored to match the border color of the corresponding image in (c).

A schematic of our AO- and 2PE-enabled 3D-STED microscope is presented in Fig. 1(a) (details in Supplement 1). Our setup is built around a custom upright microscope stand and features a 25×, 1.05 NA water-immersion objective lens with a 2 mm working distance. For depletion, we use an 80 MHz repetition rate, 775 nm pulsed laser with a pulse length of ∼600ps. To impart both the vortex and top-hat phase masks on the same depletion beam, we adopt a double-pass SLM configuration as described by Lenz *et al.* [17]. We also apply a blazed grating pattern on the SLM to isolate and block any unmodulated light.

For 2PE, we use light from a femtosecond (fs)-pulsed titanium-sapphire laser that is merged with the STED beam via a dichroic mirror. The two beams are then raster scanned across the sample by a 10 kHz resonant mirror synchronized with a galvanometric mirror. Fluorescence is collected by the same objective lens that focuses the excitation and depletion light into the sample. Red fluorescence emission wavelengths are detected in a non-descanned configuration by a photomultiplier tube (PMT), while green fluorescence emission wavelengths are descanned and directed to a SHS.

Two design features are particularly beneficial for imaging in aberrating tissue. First, we employ a water-immersion objective lens that is more closely index matched to living tissue than oil-immersion objective lenses, which thus reduces specimen-induced aberrations. Furthermore, the long 2 mm working distance of this objective lens permits long penetration depths into the specimen and accommodates a wide range of specimen dimensions and configurations. Second, we adopt an AO architecture based on WFS to correct for specimen-induced aberrations that cannot be addressed by simply matching the objective lens immersion medium to the sample. Following the developments of Wang *et al.* [39,40,43], we rely on 2PE to generate the guide star required for this WFS approach. Our correction routine is outlined in Fig. 1(b). In step (1), to generate a guide star in the sample, green fluorescent protein (GFP) or fluorescein is excited via 2PE using the same wavelength (810 nm) used to excite red fluorescence. The use of the same wavelength facilitates a rapid transition between the aberration correction and image acquisition steps. Once excited, the guide star is descanned to a SHS to generate a spot diagram, and the spot diagram is analyzed to reconstruct the aberrated wavefront. In step (2), the opposite of the measured aberration modes is added to the common beam path using a DM positioned in a plane conjugate to the back pupil of the objective lens. This aberration-correction step recovers (close to) aberration-free excitation and depletion foci at the sample. Finally, in step (3), a super-resolution image is acquired in the non-descanned red detection channel.

We note that while the green fluorescence emitted from the guide star is descanned to image the guide star onto the SHS [40], the red fluorescence channel is non-descanned since optical sectioning is inherent to 2PE and imaging the excited volume to a pinhole would be redundant. Further, in contrast to the approach by Wang *et al.*, which arranged the DM and SHS in an open-loop configuration, our implementation was inspired by the more recent implementation by Zheng *et al.* [41]. The DM and SHS in our system are arranged in a closed feedback loop, which enables measurement of any residual aberrations after correction and allows the user to optionally validate the correction and implement further rounds of correction, if necessary. This guarantees a more robust and accurate aberration-correction step.

To benchmark the resolution capabilities of our system, we imaged fixed COS7 cells featuring ATTO594-labelled microtubules or endoplasmic reticulum (ER) tubules [Fig. 1(c)]. Intensity profiles extracted from the raw tubule images were then fit using nested-loop ensemble PSF (NEP) fitting, a resolution quantification method that accounts for the underlying tubule size, which is not negligible in STED microscopy [45] (see Supplement 1). For comparison, we also acquired diffraction-limited 2PE images of the tubules. In this case, the conventional assumption that the diffraction-limited PSF is much larger than the tubule diameter is valid. Therefore, the intensity profiles extracted from these images were fit to a simple Gaussian function.

Due to the double-pass configuration of the SLM and the tunable allocation of the depletion laser power between the vortex and top-hat phase masks, lateral and axial resolution are inversely correlated in our system, i.e., allocating more laser power to the vortex phase mask to improve the lateral resolution compromises the axial resolution and vice versa. The maximum lateral resolution achievable by the system, obtained by allocating the laser power entirely to the vortex phase mask, was quantified to be 70 nm [Fig. 1(c) left; see Supplement 1). The maximum axial resolution achievable by the system using only the depletion beam component encoded with the top-hat phase mask, was quantified to be 151 nm [Fig. 1(c) right; see Supplement 1). These values are 4.2 and 6.5 times below the theoretical lateral and axial diffraction-limited resolution of our 2PE microscope, respectively (296 nm and 988 nm; NA = 1.05, refractive index = 1.33, wavelength = 810 nm) [46].

For 3D-STED imaging, it is expected that the depletion power will be distributed between the vortex and top-hat phase masks according to the resolution requirements of the given experiment. Therefore, 3D-STED resolution (in nonaberrating samples) will be a compromise between the 70 and 151 nm lateral and axial values described above [e.g., Fig. 1(c) middle].

### B. 3D-STED Microscopy of Chromatin in Mouse Skin Tissue

To demonstrate the aberration-correction capabilities of our system, we imaged epithelial nuclei in fixed mouse skin tissue. The tissue was harvested from transgenic mice expressing histone 2B-green fluorescent protein (H2B-GFP) under the control of the keratin 14 (K14) promoter. The H2B-GFP fusion proteins were then immuno-labelled with anti-GFP nanobodies conjugated to ATTO594. While the GFP fluorescence was used to generate the nonlinear guide star for aberration correction, the ATTO594 fluorescence enabled the 3D super-resolution imaging of chromatin within the epithelial nuclei.

**Fig. 2.**
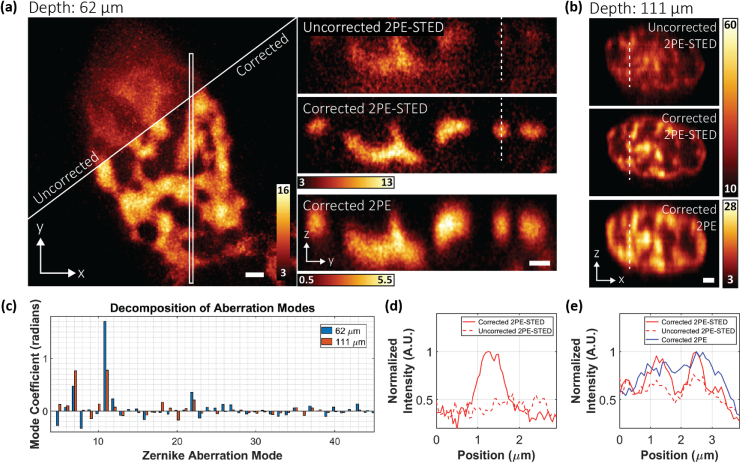
Aberration-corrected 2PE-STED microscopy in mouse skin tissue. (a) H2B-GFP distribution within the nucleus of an epithelial cell located 62 µm below the tissue surface. Left: MIP of the uncorrected (top) and corrected 2PE-STED (bottom) image stacks. Right: MIP of the region in the white box, resliced in the y−z direction from the uncorrected 2PE-STED (top), corrected 2PE-STED (middle), and corrected 2PE (bottom) image stacks. (b) MIP of uncorrected 2PE-STED (top), corrected 2PE-STED (middle), and corrected 2PE (bottom) image stacks, resliced in the x−z direction. The cell was located 111 µm below the tissue surface. In both (a) and (b), depletion via only the top-hat phase mask was used. All images were smoothed using a 3D Gaussian blur of $\sigma_{\text{xy}}=39\;nm$ and $\sigma_{z}=50\;nm$ (a) or 100 nm (b). Scale bars: 1 µm. (c) Zernike mode decomposition (modes 5 to 45) of the DM correction applied for acquiring the corrected image stacks in (a) (blue) and (b) (orange). Radians are defined with respect to λ=520nm. (d) Plot of the intensity profile at the positions marked by the white dashed lines in (a). The solid red line corresponds to the corrected 2PE-STED image. The dashed line corresponds to the uncorrected 2PE-STED image. (e) Plot of the intensity profile at the positions marked by the white dashed lines in (b). The solid red line corresponds to the corrected 2PE-STED image. The dashed red line corresponds to the uncorrected 2PE-STED image. The solid blue line corresponds to the corrected 2PE image. For (d) and (e), the profiles were acquired from a sum intensity projection of the raw, unsmoothed data.

In Fig. 2(a), we compare corrected and uncorrected (for specimen-induced aberrations) 3D-STED datasets acquired from the same volume, 62 µm deep in the labelled tissue. Instrument aberrations were corrected in both cases. For STED imaging, only the top-hat phase profile was used for depletion since the expected size scale of the higher-order chromatin organization was within the diffraction-limited lateral but not axial resolution of conventional 2PE microscopy. Since the top-hat depletion focus is more sensitive to aberrations than the vortex depletion focus, this imaging configuration was still sufficient for demonstrating the benefits of our AO approach. Indeed, the finer chromatin structures, indistinguishable without AO [Fig. 2(a), left top], were clearly resolved when aberration correction was applied [Fig. 2(a), left bottom]. This improvement is even more striking in the axial direction. We compare the uncorrected and corrected y−z images [Fig. 2(a), right] extracted from the area marked by the white box [Fig. 2(a), left]. The corrected image clearly shows details in the chromatin structures that are lost in the uncorrected image, and due to the improved axial resolution granted by the STED effect, the details no longer appear elongated as they do in the 2PE image. These effects are also illustrated by the intensity profiles in Fig. 2(d) of the areas marked by the dashed lines in Fig. 2(a). The plots show that the fluorescent signal is almost entirely absent in the uncorrected image, likely depleted by the compromised STED focus, but is recovered in the corrected image. This improvement cannot be attributed to photobleaching as the uncorrected image was acquired first.

To induce more aberrations and scattering for test purposes, a thicker skin tissue section was prepared and this time was mounted upside down so that visualization of the epithelial chromatin required aberration-corrected imaging through the adipose tissue underlying the epidermis. In this sample, our imaging volume was located 111 µm below the surface of the tissue section. Nevertheless, our AO approach still improved the aberrated image quality [Fig. 2(b), top] to a level where the chromatin structure could be clearly resolved [Fig. 2(b), middle]. Furthermore, the axial resolution was sufficient to discern details that were otherwise blurred together in the diffraction-limited 2PE image [Fig. 2(b), bottom]. These observations are confirmed by line profile intensity plots [Fig. 2(e)] measured from the areas marked by the dashed lines in Fig. 2(b).

For our AO approach, it is imperative that the acquired SHS spot diagram has a sufficient signal-to-noise level so that reliable wavefront reconstruction can be performed [Fig. 2(c); following the Zernike numbering convention of Noll [47]]. In the noninverted tissue sections, the GFP signal was sufficiently bright such that spot diagrams could be acquired in as little as 1.5 s. In the inverted skin tissue samples, we found that light scattering, likely from the adipose tissue, lowered the signal level of the spot diagrams. We therefore increased the excitation power from 3.02 to 11.29 mW and the SHS camera exposure time to 12 s to increase the detected signal.

### C. 3D-STED Microscopy of Astrocytes in Mouse Brain Tissue

To demonstrate our system’s capabilities in another aberrating sample, we imaged astrocytes in a 300 µm thick mouse brain tissue section. Glial fibrillary acidic proteins (GFAPs), markers for astrocytes, were immuno-stained with ATTO594. The labelled tissue was then mounted in fluorescein, so that a guide star could be generated from the green fluorescence using 2PE. For STED imaging, only the top-hat depletion profile was applied for axial resolution enhancement.

**Fig. 3.**
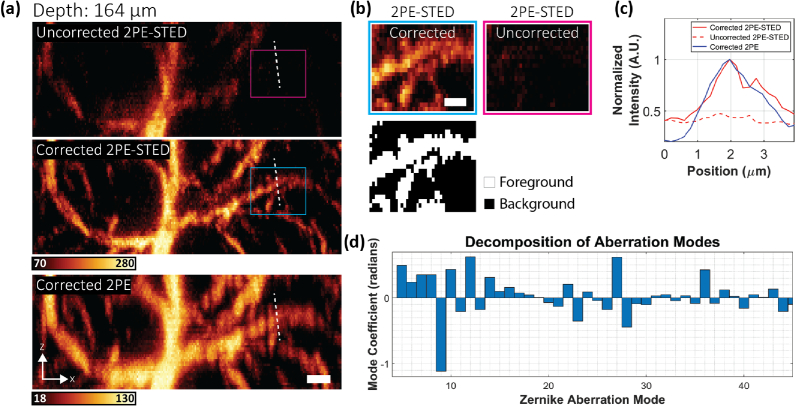
Aberration-corrected 2PE-STED microscopy in fixed mouse brain tissue. (a) MIP of uncorrected 2PE-STED (top), corrected 2PE-STED (middle), and corrected 2PE (bottom) image stacks (unsmoothed) of ATTO594-labelled astrocytes, resliced in the x−z direction. The center of the image stack was 164 µm below the tissue surface. Scale bar: 2 µm. (b) Top: Areas corresponding to the magenta and cyan boxes in (a) showing an ∼8.8-fold increase in foreground intensity between the uncorrected and corrected images. Bottom: Mask used to delineate the foreground and background pixels. The mask was generated using the Otsu method. The intensity of each image was calculated as the mean of the foreground pixels minus the mean of the background pixels. Scale bar: 1 µm (c) Plot of the intensity profiles at the positions marked by the white dashed lines in (a). The solid red line corresponds to the corrected 2PE-STED image. The dashed line corresponds to the uncorrected 2PE-STED image. The solid blue line corresponds to the corrected 2PE image. The profiles were acquired from a sum intensity projection of the raw, unsmoothed data. (d) Zernike mode decomposition of the DM correction applied for acquiring the corrected image stacks in (a).

In Fig. 3(a), uncorrected and corrected 2PE-STED and corrected 2PE x−z frames are shown from an imaging volume located 164 µm below the tissue surface (see also Visualization 1). Aberrations had a detrimental effect on the intensity and resolution of the uncorrected image. However, aberration correction [Fig. 3(d)] improved this such that the average intensity within the cyan and magenta boxes increased by a factor of ∼8.8 [Fig. 3(b)]. This effect is also exhibited in the line profile intensity plots [Fig. 3(c)]. The plots also show that two astrocyte branches that appear as one in the 2PE image are distinguishable in the corrected 2PE-STED image, elucidating the STED effect.

### D. Aberration-Corrected 2PE-STED Imaging of Neurons in a Living Mouse

**Fig. 4.**
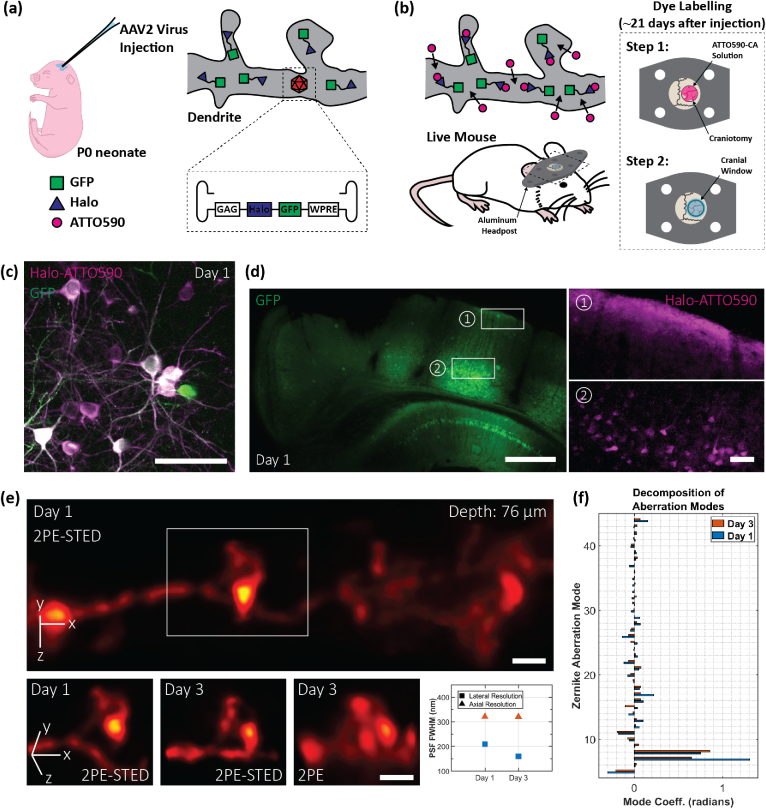
2PE-STED imaging of dendritic spines *in vivo*. (a) and (b) Strategy for neuron labelling in a living mouse using ATTO590. (c) *In vivo* 2PE overview image acquired on a commercial 2PE system 1 day after labelling. Green and magenta correspond to GFP and ATTO590 signal, respectively. Scale bar: 50 µm. (d) Wide-field images demonstrating the spatial extent of the rAAV infection (represented by GFP labelling; left), and ATTO590 labelling (right). ATTO590 images correspond to the regions outlined by white boxes in the GFP image. Scale bars: 500 µm (left) and 50 µm (right). (e) Top: Aberration-corrected STED image stack of a dendrite 76 µm below the cortical surface. Depletion via both the vortex and depletion phase masks was used. A 3D Gaussian blur of $\sigma_{\text{xy}}=60\;nm$ and $\sigma_{z}=75\;nm$ was applied. Scale bar: 1 µm. Bottom: Repeated imaging of the dendritic spine highlighted by the white box in (e). Aberration-corrected imaging was performed 1 day (first panel) and 3 days (second and third panels) days after the labelling. Scale bars: 1 µm. Resolution values, as quantified using NEP fitting, are shown in the fourth panel. Blue and orange markers correspond to days 1 and 3, respectively. On day 1, lateral and axial PSF FWHMs were 209 and 321 nm. On day 3, they were 160 and 320 nm. (f) Zernike mode decomposition of the DM correction applied for acquiring the image stacks in (e). Blue and orange bars correspond to days 1 and 3, respectively.

To demonstrate the full potential of our super-resolution deep-tissue imaging system, we performed aberration-corrected, 3D-2PE-STED imaging in the intact brain of a living mouse. For these experiments, we required an *in vivo* labelling procedure that targeted neurons in the brain with the red live-cell compatible dye ATTO590. It has previously been shown that labelling with organic dyes emitting in the red to far-red range is advantageous for *in vivo* STED microscopy, as these dyes exhibit superior photophysical properties compared to their red fluorescent protein counterparts [48]. In line with this finding, we used a wild-type CD1 mouse line and recombinant adeno-associated virus (rAAV, serotype 2) infection to induce the expression of fused cytosolic GFP and HaloTags [49] in a subset of cortical neurons [Fig. 4(a)]. While the GFP provided the fluorescence for guide star generation, the expressed HaloTags were labelled with Halo-reactive ATTO590-chloroalkane (ATTO590-CA) for 2PE-STED imaging [Fig. 4(b)].

Two-color imaging of the brain surface with a conventional 2PE microscope, one day after labelling, revealed bright neurons in both the green and red channels [Fig. 4(c)]. Despite the nonfluorogenic nature of ATTO590, 2PE imaging with excellent signal-to-noise ratio was possible as deep as 174 µm below the cortical surface, confirming the compatibility of our labelling procedure with the imaging depths accessed by our system.

To confirm the spatial extent of neuronal labelling, we performed wide-field imaging of fixed coronal brain sections, harvested 1 day after labelling [Fig. 4(d)]. rAAV infection, indicated by the GFP signal [Fig. 4(d), left], was evident throughout the injected hemisphere, and the topical application of ATTO590-CA was sufficient to label neuronal cell bodies down to ∼650µm below the brain surface [Fig. 4(d), right].

Next, we imaged ATTO590-labelled neurons in anesthetized mice with our 3D-2PE-STED instrument. In Fig. 4(e), we show an aberration-corrected 3D-2PE-STED image stack of a dendrite located 76 µm below the cortical surface (see also Visualization 2). The 3D image volume is displayed at a 70° rotation about the x axis to emphasize the 3D resolution achieved. Both the vortex and top-hat depletion phase masks were used to acquire this dataset. The white box [Fig. 4(e), top] highlights a dendritic spine that bends in and out of the x−y plane, highlighting the need for 3D imaging to capture its complete morphology [Fig. 4(e), bottom, first panel]. The same spine was re-imaged two days later [Fig. 4(e), bottom, second panel], and it exhibited morphological changes in the region of the spine head, which is possibly reconnecting with the main dendritic branch. Such details are lost when using (aberration-corrected) 2PE microscopy [Fig. 4(e), bottom, third panel] due to the inferior resolution, especially in the axial direction. Resolution quantifications are summarized in Fig. 4(e) (bottom, fourth panel).

For a typical experiment, the image volume was recorded as a set of 20µm×20µm frames (512×512 pixels and 39 nm pixel size) spaced 50 nm apart axially. Each frame took 10 s to acquire. For some areas, “jittering” artifacts could be observed that were a result of the animal’s breathing and heartbeat. If the motion was not too severe, it could be offset by offline image registration. In this case, each step in the image stack was acquired as a set of 10 individual frames, each acquired in 1 s. Each frame set was registered and averaged before being assembled into the final image stack.

However, it is preferable to mitigate the motion itself. This was achieved by positioning the coverslip sealing the craniotomy in direct contact with the brain surface, thereby suppressing tissue motion. Moreover, we avoided imaging regions near major arteries, as the blood flowing through the arteries can agitate the surrounding tissue. By observing these guidelines, motion artifacts could be sufficiently diminished such that offline image registration was not required.

In Fig. 4(f), we show the mode decomposition of the wavefront correction applied on the DM. The major corrected aberration was coma, which likely stemmed from a tilt of the cranial window. It has previously been shown that coverslip tilt can have deleterious effects on image quality by compromising the static spherical aberration correction applied via the objective correction collar [32,50]. Since it is very difficult to control, and near-impossible to eliminate window tilt during animal surgery and mounting, having a fast and adaptive means of aberration correction to compensate for this effect is crucial for 3D STED *in vivo* imaging.

## 3. SUMMARY AND OUTLOOK

Here we present a microscope that delivers subdiffraction-limit imaging resolution, in three dimensions, deep in biological tissue. We demonstrate these capabilities 164 µm deep in fixed mouse brain tissue and 76 µm deep in the brain of a living mouse. Our imaging depths were practically limited by light scattering of the fluorescence signal that was used for the SHS wavefront measurements. Further depth improvements can be envisioned by using sensorless aberration-correction approaches alone or by shifting the SHS wavefront detection wavelength into the red emission range [43]. Additional studies will also be needed to systematically determine the achievable depth-dependent resolution in different tissues.

For our *in vivo* imaging experiments, we combined the use of organic dyes, HaloTags, and rAAV technology to label neurons deep in the living mouse brain. While live-cell-compatible red organic dyes like ATTO590 offer superior STED resolution and photostability over fluorescent proteins of the same color [48], the use of rAAVs offers flexibility in the labelling scheme. The virus can be easily modified to target a different cell type, to label specific proteins (e.g., by expressing the HaloTags fused to a protein of interest), and to express additional self-labeling protein tags such as SNAP-tags so that a second live-cell-compatible dye (e.g., silicon rhodamine [48,51]) can be used for multicolor super-resolution imaging. To simplify future experiments, transgenic mouse lines that express SNAP- and/or HaloTags constitutively [48] offer a user-friendly alternative. Moreover, instead of topically applying the cell-permeable dye to the exposed brain surface, one may consider intravenously injecting dyes that are able to cross the blood-brain barrier [52]. Either or both modifications to the labelling protocol will minimize the exposure and manipulation of the imaged tissue.

It is encouraging that in our *in vivo* STED experiments we did not observe any severe structural changes in the imaged neurons that could be linked to phototoxicity. This observation may exemplify the live-cell imaging benefits of using a fast resonant scanner and redshifted excitation and depletion wavelengths as previously described [53,54]. We note here that the depletion process in a 2PE-STED system is identical to that in a conventional STED system relying on one-photon excitation. Therefore, although the study in [53] was performed on a conventional STED system, the authors’ findings regarding the potential phototoxic effects of the depletion laser and their proposed strategies for mitigating these effects are still applicable here. Nevertheless, further investigation is still required for a more detailed understanding of the potential negative effects of labeling and (2PE-)STED imaging on the physiology of the mouse brain.

The possible applications of our microscope are extensive. For example, it enables the study of neuronal plasticity in deeper layers of the brain cortex, the dynamic nanoscale organization of complex structures such as glomeruli in kidney or the structural reorganization of chromatin during cell differentiation in tissue. Furthermore, it could be combined with the super-resolution shadow imaging (SUSHI) labeling technique [55] which, by labeling the extracellular space, enables the investigation of cellular relationships and morphology in living tissue. Our technology can extend the benefits of this labelling technique deep inside the living mouse brain. Altogether, our developments represent the advancement of 3D-STED microscopy into the realm of deep-tissue (*in vivo*) imaging.

## Supplemental document

See Supplement 1 for supporting content.
