# Supplementary material for: 3D super-resolution deep-tissue imaging in living mice
Source: Optica. 2021 Mar 25;8(4):442–50. doi: 10.1364/OPTICA.416841 (PMC8243577; doi:10.1364/OPTICA.416841)
Supplement: Supplementary file 1 [file optica-8-4-442-s001.pdf]

Supplemental Document

optica

3D super-resolution deep-tissue imaging in living mice: supplement

MARY GRACE M. VELASCO,[1,2] MENGYANG ZHANG,[3,4] JACOPO ANTONELLO,[5] PENG YUAN,[4,6,7] EDWARD S. ALLGEYER,[2,8] DENNIS MAY,[9] ONS M'SAAD,[1,2] PHYLICIA KIDD,[2] ANDREW E. S. BARENTINE,[1,2] VALENTINA GRECO,[2,9,10] JAIME GRUTZENDLER,[3,4,6] MARTIN J. BOOTH,[5] AND JOERG BEWERSDORF[1,2,*]

[1]*Department of Biomedical Engineering, Yale School of Engineering & Applied Science, New Haven, Connecticut 06520, USA*
[2]*Department of Cell Biology, Yale School of Medicine, New Haven, Connecticut 06520, USA*
[3]*Interdepartmental Neuroscience Program, Yale School of Medicine, New Haven, Connecticut 06520, USA*
[4]*Department of Neuroscience, Yale School of Medicine, New Haven, Connecticut 06520, USA*
[5]*Department of Engineering Science, University of Oxford, Oxford OX1 3PJ, UK*
[6]*Department of Neurology, Yale School of Medicine, New Haven, Connecticut 06520, USA*
[7]*Current Address: Department of Biology, Stanford University, Stanford, California 94304, USA*
[8]*Current Address: The Gurdon Institute, University of Cambridge, Cambridge CB21QN, UK*
[9]*Department of Genetics, Yale School of Medicine, New Haven, Connecticut 06520, USA*
[10]*Department of Dermatology, Yale Stem Cell Center, Yale Cancer Center, Yale School of Medicine, New Haven, Connecticut 06520, USA*
[*]*Corresponding author: joerg.bewersdorf@yale.edu*

This supplement published with The Optical Society on 25 March 2021 by The Authors under the terms of the Creative Commons Attribution 4.0 License in the format provided by the authors and unedited. Further distribution of this work must maintain attribution to the author(s) and the published article's title, journal citation, and DOI.

Supplement DOI: https://doi.org/10.6084/m9.figshare.14107388

Parent Article DOI: https://doi.org/10.1364/OPTICA.416841

Supplementary Material

optica

3D super-resolution deep-tissue imaging in living mice: supplementary material

MARY GRACE M. VELASCO,[1, 2] MENGYANG ZHANG,[3, 4] JACOPO ANTONELLO,[5] PENG YUAN,[4, 6, &] EDWARD S. ALLGEYER,[2, ^] DENNIS MAY,[7] ONS M'SAAD,[1,2] PHYLICIA KIDD,[2] ANDREW E. S. BARENTINE,[1, 2] VALENTINA GRECO,[2, 7, 8] JAIME GRUTZENDLER,[3, 4, 6] MARTIN J. BOOTH,[5] AND JOERG BEWERSDORF[1, 2, *]

[1]*Department of Biomedical Engineering, Yale School of Engineering & Applied Science, CT 06520, USA*
[2]*Department of Cell Biology, Yale School of Medicine, CT 06520, USA*
[3]*Interdepartmental Neuroscience Program, Yale School of Medicine, CT 06520, USA*
[4]*Department of Neuroscience, Yale School of Medicine, CT 06520, USA*
[5]*Department of Engineering Science, University of Oxford, Oxford OX1 3PJ, UK*
[6]*Department of Neurology, Yale School of Medicine, CT 06520, USA*
[7]*Department of Genetics, Yale School of Medicine, CT 06520, USA*
[8]*Department of Dermatology, Yale Stem Cell Center, Yale Cancer Center, Yale School of Medicine, CT 06520, USA*
[&]*Current Address: Department of Biology, Stanford University, Stanford, CA 94304*
[^]*Current Address: The Gurdon Institute, University of Cambridge, Cambridge CB21QN, UK*
**Corresponding author: joerg.bewersdorf@yale.edu*

Published 25 March 2021

This document provides supplementary information to "3D super-resolution deep-tissue imaging in living mice," https://doi.org/10.1364/OPTICA.416841.

A custom-built 3D-2PE-STED microscope for aberration-corrected super-resolution imaging. A detailed schematic of our optical setup is shown in **Fig. S1**. Mechanical drawings, parts lists and software are available upon request from the authors.

Our AO-enabled 2PE-STED system features a pulsed 775 nm depletion laser with a pulse length of ~600-ps (Katana HP, OneFive). The laser beam is expanded to illuminate a spatial light modulator (SLM; X10468-02, Hamamatsu) positioned in a plane conjugate to the objective back aperture. To impart both the vortex and top-hat phase masks on the same depletion beam, we adopt a double-pass SLM configuration as described by Lenz et al. [1]. Both SLM phase masks are then imaged onto a 140-actuator deformable mirror (DM; Multi-5.5, Boston Micromachines), a 10 kHz resonance mirror (SC-30, EOPC), a galvanometric (galvo) mirror (6810P, Cambridge Technologies) and finally, the back pupil of a 25×/1.05 NA water immersion objective lens (XLPLM25XWMP2, Olympus), mounted on a custom-built upright microscope stand.

The 2PE beam is supplied by an 80 MHz fs pulsed titanium-sapphire laser (MaiTai HP, Spectra-Physics) that is merged with the STED beam via a dichroic mirror (Dichroic 1; T770dcbpxr, 5 mm thick, Chroma Technology). In the common beam path, the DM corrects for aberrations in the 2PE and STED beams simultaneously, while the resonance and galvo mirrors scan both beams along the fast and slow scanning axes, respectively. Samples were mounted on a z piezo stage (P-733.3DD, Physik Instrumente) for scanning in the axial direction.

Fluorescence emission is collected by the same objective that focuses the 2PE and STED beams into the sample. It is split from the depletion and excitation light by a dichroic mirror (Dichroic 3; T650/160dcbpxr, 5 mm thick, Chroma Technology) into two channels. In the image acquisition channel, far-red fluorescence is transmitted by Dichroic 3 and collected by a PMT (H10770PA-40-04, Hamamatsu Corporation) in a non-descanned detection geometry. The PMT is fit with two bandpass emission filters (FF01-624/40, Semrock) to isolate fluorescence emission from the organic dyes ATTO594 and ATTO590. The detected photon counts are then relayed to a pulse discriminator (F-100T, Advanced Research Instruments Corporation) and then to a custom-made circuit board for gated detection (Opsero Electronics). This board

is synchronized to the excitation laser trigger signal and it enables software control of the detection window width and delay. This same circuit board is also used to generate a trigger signal for the depletion laser such that its pulses are synchronized with those of the excitation laser. Laser blanking and galvo mirror control during image acquisition is performed by a field-programmable gate array (FPGA) board (PCIe-7841R, National Instruments), while photon counting and image formation is performed by a second FPGA board (PCIe-7852R, National Instruments). Both FPGA boards are synchronized to the motion of the resonance mirror.

In the wavefront sensing channel, fluorescence from the non-linear guide star is reflected by Dichroic 3, descanned, and then coupled out of the common beam path by another dichroic mirror (Dichroic 2; T560lpxrxt, 5 mm thick, Chroma Technology). The fluorescence is then passed through two bandpass emission filters (FF01-510/84, Semrock) and routed to a SHS, which is positioned such that the microlens array (64-483, Edmund Optics) is conjugate to the back focal plane of the objective lens. The SHS spot diagram is acquired by an EMCCD camera (iXon EM+ 897, Andor Technology) positioned at the focal plane of the microlens array and synchronized to image acquisition by the PMT. Though the wavefront sensing channel is spectrally isolated from the non-descanned imaging channel (red emission from ATTO590 or ATTO594), the same 2PE wavelength of 810 nm could be used to excite fluorescence in both channels simultaneously. This configuration enabled rapid switching between aberration correction and image acquisition. Any pre-bleaching of the red fluorophores or sample damage during wavefront sensing was not obvious during image acquisition, though additional detailed experiments would be required to quantify these effects.

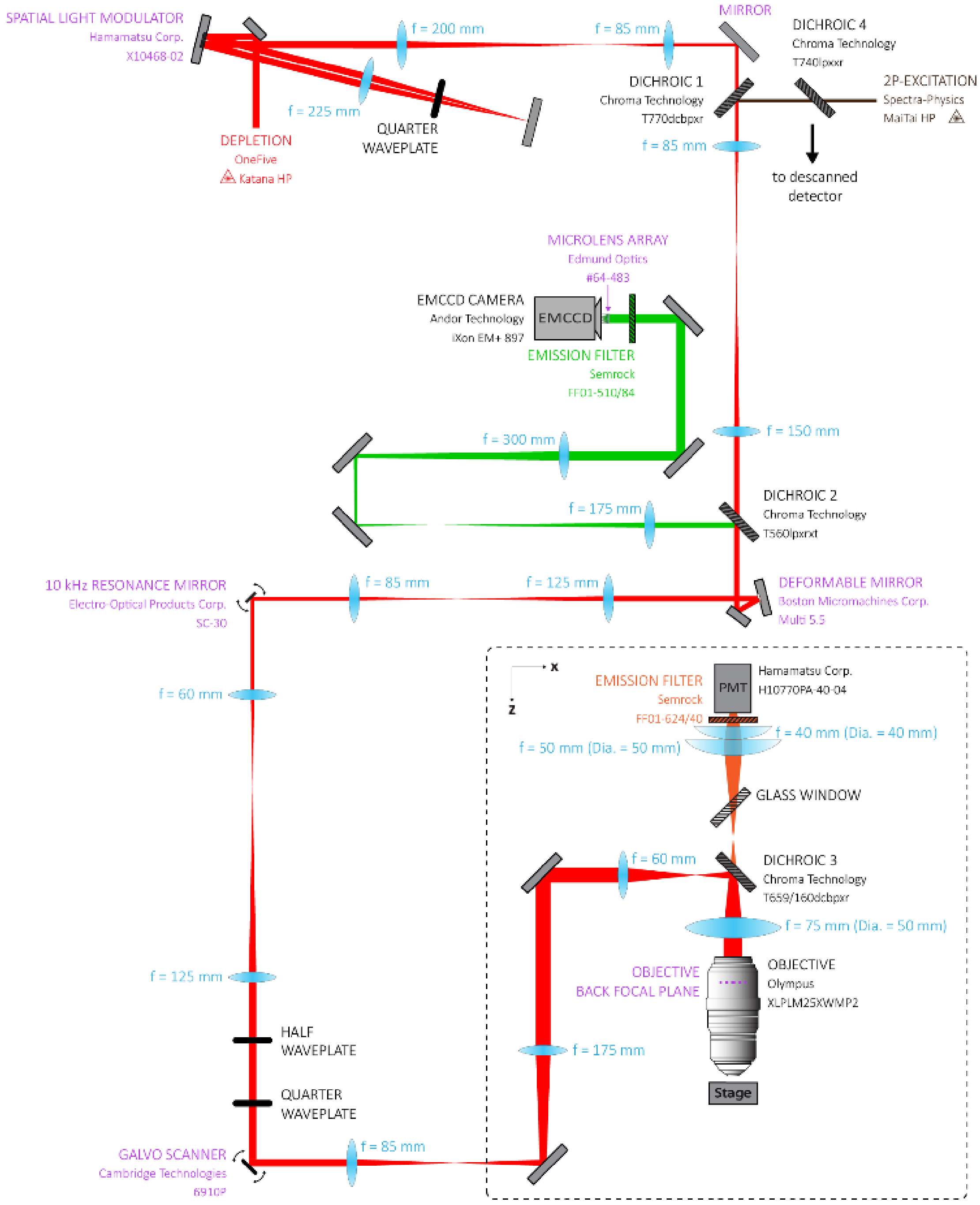

Fig. S1. Detailed schematic of our 3D-2PE-STED microscope. See text for details. All optics labeled in purple are positioned in conjugate planes. Dichroic 4 (not discussed in text) is included in the schematic for completeness but was not crucial to the experiments discussed here.

DM and SH Sensor Characterization. The preliminary calibration of the DM was performed prior to integrating the DM into the microscope system. The individual actuator influence functions were measured using an offline Michelson interferometer and decomposed into their constituent Zernike modes as described in [2]. This generated a matrix that, once inverted, could output the appropriate actuator voltage settings for each Zernike mirror mode. The shape of the DM surface could then be controlled by applying any linear combination of these modes.

Once calibrated, the DM was integrated into the microscope system, in a plane conjugate to the objective back pupil. To correct for system aberrations, we imaged 200 nm crimson fluorescent beads (F8806, Invitrogen) embedded in phosphate buffered saline (PBS) containing fluorescein (46955, Sigma-Aldrich) and used a metric-based AO approach to optimize the quality of the bead images [3]. We corrected Zernike mirror modes 4 to 11 using an image brightness metric. Next, we acquired a reference spot diagram by exciting (via 2PE) and descanning the fluorescein emission to the SHS. This reference spot diagram was then used to perform wavefront sensing, as described in [4].

In the final step, the DM was recalibrated *in situ*, using the SHS (rather than an offline interferometer) to decompose the actuator influence functions into Zernike modes. This additional calibration step, performed with the DM in the system, eliminated any discrepancy between the Zernike mode reference frame of the DM (previously defined relative to the interferometer) and that of the SHS.

Resolution Quantification. The resolution of our system was quantified as described in the main text, using the nested-loop ensemble PSF (NEP) fitting method [5] to account for underlying structure size where appropriate. To quantify the lateral resolution from $STED_{xy}$ images of microtubules (as shown in Fig. 1(c), left), standard NEP fitting with a Lorentzian-PSF microtubule model function was used. To quantify the lateral and axial resolution from 3D-STED and $STED_z$ image stacks (as shown in Fig. 1(c), middle and right, and Fig. 4(e)), the NEP fitting procedure was extended to quantify the axial PSF size in addition to the lateral PSF and feature sizes.

Table S1. Line Profile Parameters

Figure	Imaging Mode	Structure Imaged	Line Profile Widths (nm)	# of FOVs	# of Profiles	PSF FWHM (nm)	Mean Structure Diameter ± Standard Deviation (nm)
1(c), left	$STED_{xy}$	Microtubules	w_{xy} = 1,955*<br>w_z = N/A	8	N = 105	PSF_{xy} = 70<br>PSF_z = N/A	N/A
1(c), middle	3D-STED	ER tubules	w_{xy} = 391<br>w_z = 400	1	N = 70	PSF_{xy} = 133<br>PSF_z = 454	107 ± 28
1(c), middle	2PE	ER tubules	w_{xy} = 391<br>w_z = 400	1	N = 70	PSF_{xy} = 354<br>PSF_z = 1,131	N/A
1(c), right	$STED_z$	ER tubules	w_{xy} = 391<br>w_z = 400	7	N = 160	PSF_{xy} = 247<br>PSF_z = 151	112 ± 28
4(e), Day 1	3D-STED	Neuron branches	w_{xy} = 586<br>w_z = 600	1	N = 12	PSF_{xy} = 209<br>PSF_z = 321	366 ± 48
4(e), Day 3	3D-STED	Neuron branches	w_{xy} = 586<br>w_z = 600	1	N = 7	PSF_{xy} = 160<br>PSF_z = 320	407 ± 47

*average width of hand-drawn line profiles

Multi-axis (lateral and axial) line profiles were extracted such that they intersected at the 3D center of each tubule cross-section. This allowed the algorithm to fit a single tubule diameter shared by both profiles. As a result, the total number of fit parameters that determined the model widths was reduced from 4N to N + 2, where N is the number of line profiles. First, lateral profiles were hand-drawn on an axially-summed or maximum-intensity-projected two-dimensional (2D) image using FIJI. The center positions, angles, and lengths of these profiles were then saved and imported by a Python script. For each manually drawn profile, a preliminary lateral profile was extracted from the axially summed image stack and fit with a Gaussian to determine the lateral coordinates of the tubule center position. Next, a square grid, sized $w_{xy} \times w_{xy}$ (see **Table S1**) with spacings matching the image pixel size (see **Table S2**), was generated about this center position and rotated to match the line orientation. Each slice of the image stack was then interpolated using a bivariate spline of order 3 in both x and y, sampled using the square grid, and the average value from this interpolation was taken to be the axial profile intensity value at that slice. The extracted axial profile was then fit to a Gaussian to

determine the tubule center along *z*. A linear interpolation of the data was sampled over a depth of w_z (see **Table S1**) centered on the tubule. This interpolated ministack was then averaged axially to create a 2D image from which the final lateral profile of width w_{xy} could be extracted.

NEP fitting with a Lorentzian PSF model was used for all STED resolution quantifications. Constant background terms were included in the model, with an additional linear background term in z for ensemble fitted profiles to account for any drop in intensity vs. z-stack slice number. For 2PE resolution quantification, lateral and axial line profiles were extracted as described above, but were fit to a simple Gaussian function. NEP fitting was no longer required since the (close to) diffraction-limited PSF was much larger than the underlying structure size in all three dimensions. In our 2PE data, the simple Gaussian fits resulted in a 30% lower mean mean-squared error than simple Lorentzian fits, indicating that the Gaussian is a better model of our 2PE PSF, as expected.

Imaging Parameters. Acquisition parameters for all images presented in the main text are summarized in Table S2. All images, except for Fig. 4(c) and 4(d), were acquired using our custom 3D-STED setup using an excitation wavelength of 810 nm and a depletion wavelength of 775 nm. Fig. 4(c) was acquired on a 2PE microscope (Ultima Investigator, Bruker Corporation) with multi-color imaging capabilities. An excitation wavelength of 810 nm was also used. Fig. 4(d) was acquired on a widefield microscope (DM IL LED, Leica Microsystems), equipped with a CCD camera (DR-328G-C01-SIL Clara, Andor Technology).

Table S2. Imaging Parameters

Figure	Depth (µm)	Line Accumulations	FOV size[&] (pixels) (µm)	Voxel size (nm)	810 nm Exc. Power (mW)	775 nm Depl. Power (mW)
1(c) Left	-	2PE: 100<br>STED: 400	1024 × 1024 × N/A<br>20 × 20× N/A	19.5 × 19.5 × N/A	5.20	Not measured
1(c) Middle	-	2PE: 300<br>STED: 600	512 × 512 × 40<br>20 × 20 × 2	39 × 39 × 50	2.30	Total: 216
1(c) Right	-	2PE: 300<br>STED: 200	256 × 256 × 50<br>20 × 20 × 2	78 × 78 × 40	2.17	Top-hat:
2(a)	62	STED: 200	512 × 512 × 120<br>20 × 20 × 6	39 × 39 × 50	2PE image: 0.85<br>STED image: 1.88	Top-hat: 98
2(b)	111	2PE: 100<br>STED: 400	512 × 512 × 80<br>20 × 20 × 8	39 × 39 × 100	3.02	Not measured
3(a)	164	2PE: 150<br>STED: 500	256 × 256 × 50<br>30 × 30 × 10	117 × 117 × 200	Not measured	Not measured
4(c)	104	-	1024 × 1024 × 40<br>292 × 292 × 40	286 × 286 × 1000	Not measured	Not measured
4(e)	76	See text above	512 × 512 × 80<br>20 × 20 × 4	39 × 39 × 50	2PE image: 1.92<br>STED image: 10.60<br>(Day 3)	Total: 160<br>(Day 3)

[&]refers to original uncropped image

The specified imaging depth is measured from the surface of the tissue section or brain to the center of the 3D image stack. "Line Accumulations" refers to the number of times each line in the frame was scanned by the resonance scanner. Fluorescence was only recorded on the forward scan of the mirror. For the image stack in **Fig. 4(e)**, motion artifacts as described in the main text, were prevalent in our initial images. To mitigate this effect, each step in the stack was acquired as a set of 10 individual frames, with the line accumulations set to 20 lines for each frame. Each set of frames was then registered and averaged before being assembled into the final image stack. Excitation and depletion laser powers were measured at the sample, using a power meter (PM100D, Thorlabs) with a photodiode or thermal (S170C or S175C, Thorlabs) sensor.

Aberration-correction Routine. For all aberration-corrected imaging, spot diagram acquisition was performed prior to image acquisition by scanning the non-linear guide star across a single 2D plane in the middle of the imaged volume. Spot diagrams were analyzed using an algorithm described in [4]. As a minor

modification, we localized each spot in the spot diagram using a radial symmetry centers approach [6] rather than centroiding. The calculated phase correction was then applied to the DM for the entire acquisition of the image stack.

The SHS EMCCD exposure times ranged between 1.5 s for the brightest samples and 12 s, if scattering was an issue. However, most spot diagram acquisitions required 5 s exposure times or less. The excitation laser power during spot diagram acquisition were set to be the same as during STED image acquisition, except for the image in Fig. 2(b) where the excitation power was increased from 3.02 mW to 11.29 mW for correction.

In our experiments, we found that it was sufficient to qualitatively gauge the success of the aberration-correction routine based on the image quality of the final corrected image. However, should stricter conditions and/or further rounds of correction be required, a convenient parameter for quantifying the success of each correction round is the root-mean-square of the remaining wavefront deviation. After each correction, this is easily calculated as the square root of the sum of the squares of the residual Zernike aberration mode coefficients [4].

Cell Culture and Labelling. An 18 × 18 mm coverslip was sonicated for 15 minutes in potassium hydroxide, rinsed three times with Milli-Q water, sterilized with 100% ethanol, incubated with poly-l-lysine for 10 minutes, and then rinsed once with phosphate-buffered saline (PBS).

For endoplasmic reticulum (ER) labelling, COS7 cells were electroporated with an ER membrane marker (mEmerald-Sec61-C-18, a gift from Michael Davidson, Addgene plasmid #54249) before they were seeded on the coverslip and grown overnight. The cells were fixed with 3% paraformaldehyde (PFA; 15710, Electron Microscopy Science) + 0.1% glutaraldehyde (GA; 16019, Electron Microscopy Science) diluted in PBS that was pre-warmed to 37°C for 15 minutes. The cells were rinsed three times in PBS, permeabilized (PBS + 0.05% IGEPAL CA-630 + 0.05% Trition X-100 + 0.1% Bovine Serum Albumin) for 3 minutes, and then rinsed an additional three times with PBS. The cells were incubated in block buffer (PBS + 0.05% IGEPAL CA-630 + 0.05% TritionX-100+ 5% normal goat serum) for 1 hour then incubated with rabbit anti-GFP antibodies (1:500 dilution in block buffer; A-11122, Invitrogen), overnight at 4°C. The cells were rinsed three times for five minutes each with wash buffer (PBS + 0.05% IGEPAL CA-630 + 0.05% Trition X-100+ 0.2% Bovine Serum Albumin), incubated with secondary anti-rabbit antibodies conjugated to ATTO594 (1:1000 dilution in block buffer; 77671, Sigma-Aldrich) for an hour, and then washed three more times for five minutes each. The cells were post fixed with 3% PFA + 0.1% GA diluted in PBS for 10 minutes, rinsed 3 times with PBS, then mounted on a microscope slide using ProLong Diamond Antifade Mountant (P36961, Invitrogen).

For microtubule labelling, COS7 cells were incubated with 0.2% saponin diluted in cytoskeletal buffer (CBS; 10 mM MES pH 6.1, 138 mM NaCl, 3 mM MgCl2, 2 mM EGTA, 320 mM sucrose) that was warmed to 37 °C for 1 minute. The cells were fixed with 3% PFA + 0.1% GA diluted in CBS warmed to 37 °C for 15 minutes, then rinsed 3 times with PBS. Cells were blocked and permeabilized with 3% bovine serum albumin (BSA; 001-000-162, Jackson ImmunoResearch) and 0.2% Triton X-100 (T8787, Sigma-Aldrich) diluted in PBS for 30 minutes. The cells were incubated with mouse anti-α-tubulin antibody (T5168, Sigma-Aldrich) diluted to 1:200 in antibody dilution buffer (1% BSA and 0.2% Triton X-100 in PBS) overnight at 4 °C and then washed three times for five minutes each with wash buffer (0.05% Triton X-100 in PBS). Next, the cells were incubated with anti-mouse secondary antibodies conjugated to ATTO594 (76085-1ML-F, Sigma-Aldrich) for 1 hour and then washed three times for five minutes each with wash buffer. The cells were then post fixed with 3% PFA + 0.1% GA, diluted in CBS for ten minutes and then rinsed three times with PBS. Finally, the cells were mounted in on a microscope slide using ProLong Diamond Antifade Mountant.

Mouse Skin Tissue Preparation and Labelling. Skin tissue was harvested from a transgenic mouse expressing histone 2B-green fluorescent protein (H2B-GFP) under the control of the keratin 14 (K14) promoter. The tissue was dissected and incubated with 3.5 mg/mL Dispase II (4942078001, Sigma-Aldrich) in PBS for 10 minutes at 37°C. The epidermis was then peeled off of the dermis with fine forceps and fixed in 4% PFA in PBS for 10 minutes. Next, the fixed tissue was incubated in blocking solution (5% normal goat serum, 1% BSA, and 0.2% Triton X-100 in PBS) for 30 minutes, then incubated with anti-GFP nanobodies conjugated to ATTO594 (diluted 1:200 in blocking solution; gba594, ChromoTek) overnight at 4°C. The tissue was then washed in blocking solution 3 times for 10 minutes each, then mounted in ProLong Diamond Antifade Mountant.

For thick tissue preparations, tissue was harvested and dissected as described above, then immediately fixed in 4% PFA in PBS for 1 hour at room temperature. The fixed tissue was then incubated in blocking solution for 6-8 hours at room temperature on a rocking platform. Next, the blocked tissue was incubated with ATTO594-conjugated anti-GFP nanobodies (prepared as described above) for ~66 hours at room temperature, washed in PBS with 2% Triton X-100 for 6-8 hours, and mounted upside-down (i.e. with the skin surface positioned face-down) in ProLong Diamond Antifade Mountant for imaging.

Mouse Brain Tissue Preparation and Labelling. A whole mouse brain, fixed in 4% PFA overnight, was sectioned into 300 μm slices using a vibratome (VT1000 S, Leica Biosystems). The sections were then placed in permeabilization buffer (0.5% Triton X-100 in 1× PBS) for 1 hour on a rocking platform at room temperature. Next, the sections were stained with a GFAP polyclonal primary antibody (1:500 dilution; ab7260, Abcam) in antibody dilution buffer (1% BSA + 0.2% Triton X-100 in 1× PBS) first for 24 hours at room temperature on a rocking platform and then for 5 days at 4°C. The sections were subsequently stained with an ATTO594-conjugated secondary antibody (1:500 dilution; 77671, Sigma-Aldrich) for 72 hours at 4°C. After each staining step, the sections were washed 5-7 times with wash buffer (0.05% Triton X-100 in 1× PBS) for 30 minutes each. The samples were then rinsed with PBS and mounted in fluorescein (46955, Sigma-Aldrich) in PBS.

Animals. All animal procedures described have been approved by the Institutional Animal Care and Use Committee at Yale University. CD1 Mice were purchased from Charles River.

Plasmid Generation and Viral Vector Production. The AAV-CAG-Halo-GFP viral vector was constructed from the AAV-CAG-GFP plasmid (Addgene plasmid #28014). The GFP sequence was replaced with a Halo-GFP sequence.

Recombinant adeno-associated viruses (rAAVs) of serotype 2 were produced and purified following procedures described previously [7], using a two-plasmid helper free system (PlasmidFactory). HEK293T cells were co-transfected with the transfer plasmid, containing the target gene, and the helper plasmid, using JetPrime reagents. Transfected cells were collected 4-5 days later. Virus particles were extracted from the cell lysate

and purified by iodixanol gradient ultracentrifugation and titered by transfection assay.

Neonatal Injection of rAAVs. rAAV viruses were injected into the subarachnoid space of P0 CD1 mice, using a Picospritzer microinjection system (051-0500-900, Parker Hannifin Corporation). Injection pipettes were prepared from glass capillary tubes (3-000-210-G, Drummond Scientific) using a pipette puller (PC-10, Narishige), loaded with the prepared virus solution, and connected to the Picospritzer pipette holder. To prepare for the injection, the mouse pups were placed on ice to induce anesthesia via hypothermia. Next, approximately 1 µl of virus solution was injected into the subarachnoid space of each pup. After the injection, the pups were placed on a warming pad until their body temperature and skin color returned to normal and they began to move. The total number of virus particles injected into a single mouse was approximately 10E7.

ATTO590 Dye Labelling and Installation of Cranial Window. This procedure was performed at least 21 days after the neonatal injection of rAAVs, and only if the animal recovered normally. The infected mouse was weighed and dosed with buprenorphine (0.1 mg/kg) and ketamine/xylazine (K/X; 100 mg/kg Ketamine, 10 mg/kg Xylazine) via intraperitoneal injection. Furthermore, dexamethasone (2mg/kg) and carprofen (5 mg/kg) were administered via subcutaneous injection. When the mouse was no longer responsive to a firm toe pinch, ophthalmic ointment was applied, the head was shaved and the exposed skin was treated with povidone-iodine solution and cleaned with ethanol. A small piece of skin was then removed to expose the skull, and the membrane tissue covering the skull surface was removed using forceps. The animal was restrained onto a custom stage mount fitted with a heated pad. Next, a dental drill was used to create a 3 mm diameter craniotomy, centered ~2.5 mm from both Bregma and midline. During this step, the skull was periodically rinsed with sterile PBS to prevent excessive heating. Once the skull was sufficiently thinned, the circular piece of skull was gently lifted and removed using forceps. GELFOAM Sterile Sponge (0315-08, Pfizer Inc.) was used to absorb any bleeding. Next, fine forceps and a needle were used to pierce the dura and gently pull it to one side. 25 µl of ATTO590-CA dye solution was then applied topically onto the exposed brain surface. To prepare the dye solution, 8 µl of 0.6 mM ATTO590-CA solution (molecular weight = 788 g/mol; resuspended in DMSO) was mixed with 40 µl of sterile PBS and 2 µl of Pluronic for a final ATTO590-CA concentration of 96 µM. After incubating for 30 minutes, the area was washed with sterile PBS and the craniotomy was sealed with a piece of #1.5 cover glass, 3 mm in diameter. Two-component silicone putty (Picodent Twinsil, Picodent) was then applied to the circular opening of the head post to protect the area. The animal was then released and allowed to recover in a cage positioned above a heated pad. For animals that underwent multiple imaging sessions over days, buprenorphine was administered twice daily and carprofen (5 mg/kg) was administered once daily for the 2 days following the procedure.

***in vivo* Imaging.** The animal was anesthetized using ketamine/xylazine (100 mg/kg Ketamine, 10 mg/kg Xylazine) via intraperitoneal injection., and when the mouse was no longer responsive to a firm toe pinch, it was restrained onto the custom stage mount via the surgically installed head post. The two-component silicone putty (Picodent Twinsil, Picodent) protecting the circular opening of the head post was removed to expose the glass window, and the window was cleaned with sterile PBS to remove any residue. The custom stage base holding the mouse was then mounted onto the 2PE-STED microscope for *in vivo* super-resolution imaging.

At the end of the imaging session, if the animal was to undergo additional imaging experiments, it was released from the stage mount and allowed to recover in its cage, which was positioned over a heating pad. Otherwise, the mouse was euthanized, either via cardiac perfusion or cervical dislocation.

References

1. M. O. Lenz, H. G. Sinclair, A. Savell, J. H. Clegg, A. C. Brown, D. M. Davis, C. Dunsby, M. A. Neil, and P. M. French, "3-D stimulated emission depletion microscopy with programmable aberration correction," J. Biophotonics **7**, 29-36 (2014).
2. J. Antonello, T. van Werkhoven, M. Verhaegen, H. H. Truong, C. U. Keller, and H. C. Gerritsen, "Optimization-based wavefront sensorless adaptive optics for multiphoton microscopy," J. Opt. Soc. Am. A **31**, 1337-1347 (2014).
3. D. Debarre, E. J. Botcherby, T. Watanabe, S. Srinivas, M. J. Booth, and T. Wilson, "Image-based adaptive optics for two-photon microscopy," Opt. Lett. **34**, 2495-2497 (2009).
4. J. Antonello, "Optimisation-based wavefront sensorless adaptive optics for microscopy," (Delft University of Technology, 2014).
5. A. E. S. Barentine, L. K. Schroeder, M. Graff, D. Baddeley, and J. Bewersdorf, "Simultaneously measuring image features and resolution in live-cell STED images," Biophys. J. **115**, 951-956 (2018).
6. R. Parthasarathy, "Rapid, accurate particle tracking by calculation of radial symmetry centers," Nat. Methods **9**, 724-726 (2012).
7. D. Grimm, M. A. Kay, and J. A. Kleinschmidt, "Helper virus-free, optically controllable, and two-plasmid-based production of adeno-associated virus vectors of serotypes 1 to 6," Molecular Therapy **7**, 839-850 (2003).

Visualization 1. Aberration-corrected 2PE-STED imaging of ATTO594-labelled astrocytes in fixed mouse brain tissue. Related to Fig. 3.

Visualization 2. Aberration-corrected 2PE-STED imaging of ATTO590-labelled neurons in a living mouse. Related to Fig. 4.
